# Pulmonary Pathogens Adapt to Immune Signaling Metabolites in the Airway

**DOI:** 10.3389/fimmu.2020.00385

**Published:** 2020-03-13

**Authors:** Sebastián A. Riquelme, Tania Wong Fok Lung, Alice Prince

**Affiliations:** Department of Pediatrics, Columbia University Medical Center, New York, NY, United States

**Keywords:** *Pseudomonas aeruginosa*, *Staphylococcus aereus*, succinate, fumarate, cystic fibrosis, COPD, immunometabolism, inflammation

## Abstract

A limited number of pulmonary pathogens are able to evade normal mucosal defenses to establish acute infection and then adapt to cause chronic pneumonias. Pathogens, such as *Pseudomonas aeruginosa* or *Staphylococcus aureus*, are typically associated with infection in patients with underlying pulmonary disease or damage, such as cystic fibrosis (CF) or chronic obstructive pulmonary disease (COPD). To establish infection, bacteria express a well-defined set of so-called virulence factors that facilitate colonization and activate an immune response, gene products that have been identified in murine models. Less well-understood are the adaptive changes that occur over time *in vivo*, enabling the organisms to evade innate and adaptive immune clearance mechanisms. These colonizers proliferate, generating a population sufficient to provide selection for mutants, such as small colony variants and mucoid variants, that are optimized for long term infection. Such host-adapted strains have evolved in response to selective pressure such as antibiotics and the recruitment of phagocytes at sites of infection and their release of signaling metabolites (e.g., succinate). These metabolites can potentially function as substrates for bacterial growth and but also generate oxidant stress. Whole genome sequencing and quantified expression of selected genes have helped to explain how *P. aeruginosa* and *S. aureus* adapt to the presence of these metabolites over the course of *in vivo* infection. The serial isolation of clonally related strains from patients with cystic fibrosis has provided the opportunity to identify bacterial metabolic pathways that are altered under this immune pressure, such as the anti-oxidant glyoxylate and pentose phosphate pathways, routes contributing to the generation of biofilms. These metabolic pathways and biofilm itself enable the organisms to dissipate oxidant stress, while providing protection from phagocytosis. Stimulation of host immune signaling metabolites by these pathogens drives bacterial adaptation and promotes their persistence in the airways. The inherent metabolic flexibility of *P. aeruginosa* and *S. aureus* is a major factor in their success as pulmonary pathogens.

## Introduction

*Pseudomonas aeruginosa* and *Staphylococcus aureus* are major respiratory pathogens that activate airway inflammation to produce pneumonia. These pathogens express a number of specific virulence determinants that can directly damage host tissues. They also activate host inflammation that contributes to the damage via the release of proteases and ROS by recruited immune cells. Such opportunistic pathogens are common in the environment and when inadvertently inhaled are usually cleared by normal mucociliary function. An excessive inflammatory response and/or and especially virulent organisms result in the fulminant acute pneumonias with high mortality, as described for the toxin-producing methicillin-resistant *S. aureus* (MRSA) (1) and *P. aeruginosa* (2). More commonly, these pathogens adapt to the milieu within the airway and cause a more chronic infection. In individuals with genetic or acquired respiratory dysfunction, such as in cystic fibrosis (CF) and chronic obstructive pulmonary disease (COPD), these bacteria are able to colonize the respiratory tract, adapt to the milieu of the airway by altering their metabolic activity and through the selection of mutants that have enhanced fitness in the presence of inflammatory products and metabolites. Both *S. aureus* and *P. aeruginosa* undergo a gradual adaptation to the human airway, resulting in the production of biofilms that thwart opsonization by antibody or complement, inhibit phagocytosis, and create a barrier for antibiotic penetration. Although the mechanisms used by these pathogens to attach to the lung parenchyma and the genetics of their subsequent biofilm formation are well-known, exactly what triggers this adaptation remains less clear. In this review we discuss how *P. aeruginosa* and *S. aureus* have evolved mechanisms to adapt to the airway environment to cause acute and chronic lung infections.

## Acquisition of *P. Aeruginosa* From the Environment

*P. aeruginosa* are highly versatile opportunists with a large genome and tremendous metabolic flexibility (3). These organisms normally reside in an aquatic environment, in streams, soil, plants and readily contaminate hospital paraphernalia. *P. aeruginosa* enjoys genetic adaptability and readily acquires novel genes in response to selective pressure, such as those conferring antimicrobial resistance determinants as well as altering expression of its own gene products, such as its chromosomal β-lactamase *ampC*, as a response to antibiotics in the environment. This genetic flexibility has led to multi-drug resistance and designation by the CDC and WHO as an exceptionally important human pathogen (4). Within its large genome, *P. aeruginosa* retains numerous genes that are activated specifically in response to contact with eukaryotic hosts, and alter expression of numerous metabolic and secreted proteins. Some of these changes in gene expression are in direct response to host immune pressure, either to avoid phagocytic clearance or to exploit and respond to immune cell products.

The pathogenesis of airway infection by *P. aeruginosa* has been well-studied, driven in part by the unusual association of this specific pathogen with cystic fibrosis (5), infecting over 75% of CF patients and contributing substantially to their pulmonary disease (6). The longitudinal study of *P. aeruginosa* strains from CF patients over decades has provided a wealth of information detailing the geno-phenotypic adaptation of these organisms to the human lung and has been highly relevant to the pathogenesis of other airway opportunists. The organisms inadvertently inhaled from a contaminated environment express a number of gene products to initiate infection: flagella for bacterial motility, pili for attachment, siderophores to trap iron and micronutrients as well as proteases and toxins that generate substrates for bacterial growth (5). Each of these has associated immunogenicity and is recognized by a specific pattern recognition receptor that initiates host airway inflammation. Substantially different genes are activated later *in vivo* in response to the local milieu in the airway, particularly the presence of immunometabolites.

## Innate Immune Responses to *P. aeruginosa*

### LPS Induces the Release of Succinate, Activation of Inflammasomes, and IL-1β

**Figure 1:**
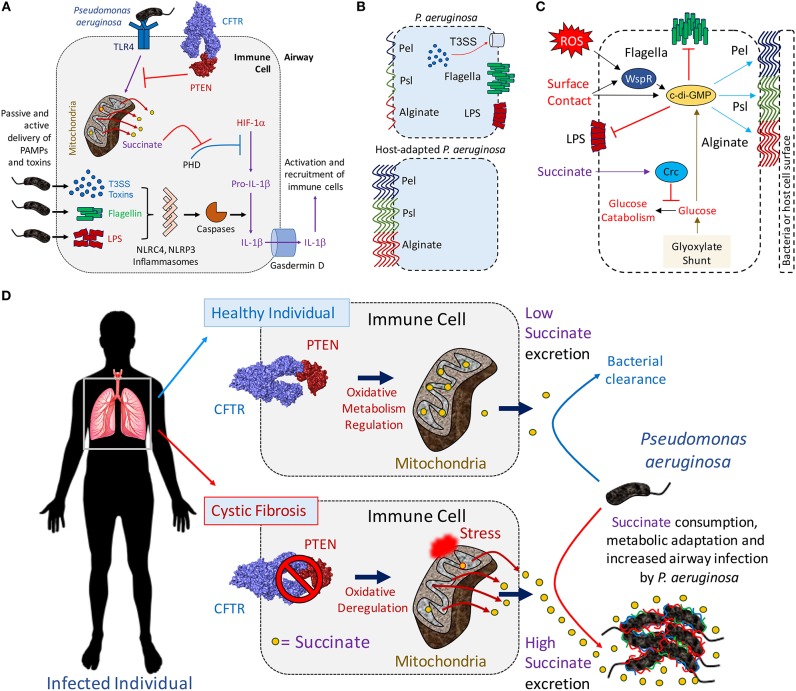
The mitochondrial PTEN-succinate axis promotes *P. aeruginosa* airway infection. **(A)** Once interacting with surface TLR4 receptor, *P. aeruginosa* LPS triggers inflammation. Mitochondria become depolarized producing ROS and succinate (yellow circles). This is a tightly regulated process by the CFTR-PTEN complex, which signals through Akt and PI3Kγ/δ to suppress LPS-TLR4-driven inflammation. Succinate leaked into the cytoplasm inhibits PHD, which activates HIF-1α to induce production of pro-IL-1β. In parallel, *P. aeruginosa* release soluble LPS in the cytoplasm, flagellin or inject T3SS toxins. These activate different inflammasomes, priming caspases and cleavage of pro-IL-1β to produce IL-1β. Gasdermin D form pores in the cell membrane releasing mature IL-1β and causing cell death. Once extracellular, IL-1β induces recruitment of more immune cells to clear infection. **(B)** Laboratory and *P. aeruginosa* clinical strains derived from acutely infected patients (e.g., ICU) are more immunostimulatory than host-adapted isolates, such as in CF. **(C)** The immunosignaling metabolite succinate induces *crc*-dependent polysaccharide production by repressing glucose catabolism in *P. aeruginosa*. Extracellular ROS and surface contact also induce c-di-GMP generation, which promotes the synthesis of Pel, Psl and alginate and biofilm production. The pro-gluconeogenic glyoxylate shunt pathway contributes with extracellular polysaccharides production by shunting carbon atoms into glucose synthesis. **(D)** Insufficient CFTR-PTEN interaction induces excessive succinate release (yellow circles) by immune cells and *P. aeruginosa* airway adaptation. In healthy individuals (upper panel), the CFTR-PTEN complex regulates the oxidative state of mitochondria. In cystic fibrosis patients (lower panel), lack of membrane CFTR-PTEN induces mitochondrial deregulation, producing ROS and more succinate release. Cytoplasmic succinate is excreted into the extracellular milieu feeding *P. aeruginosa*. Succinate-stressed *P. aeruginosa* upregulate anti-oxidant genes, causing metabolic adaptation, extracellular polysaccharide synthesis (color lines) and biofilm production. Biofilm-producing *P. aeruginosa* attach to the airway parenchyma, which protects them from phagocytes, antibiotics and antibodies.

LPS, lipopolysaccharide or endotoxin, is a major component of *P. aeruginosa* that activates immune signaling (7). Upon recognition of LPS by toll-like receptor 4 (TLR4), resident alveolar macrophages and neutrophils increase glycolysis, succinate oxidation and generation of reactive oxidative species (ROS) (8–10) (Figure 1A). When primed by LPS, mitochondria shunt succinate into the cytoplasm, which inhibits prolyl-hydroxylase activity (PHD) and enable the stabilization of HIF-1α (11) (Figure 1A). HIF-1α, in turn, induces expression of pro-IL-1β mRNA (8, 11). This transcript is translated into the inactive pro-IL-1β form, which by the action of caspases is cleaved. Mature IL-1β is then released into the extracellular milieu where it activates local and surveilling phagocytes that sense and increase their bactericidal activities.

These immune responses are generated by inflammasome activation. Inflammasomes are complexes of multiple proteins that function together to transduce the signals provided by K^+^ fluxes, specific microbial gene products or DNA into the activation of pro-caspases (12). While the NLRC4 inflammasome recognizes *P. aeruginosa* type 3 secretion system (T3SS) toxins and flagellin monomers, NLRP3 senses cytoplasmic LPS (13–15). Through the activity of caspase-1, the substrate gasdermin D is cleaved and produces membrane pores resulting in a form of cell death termed pyroptosis and the leakage of intracellular contents, such as metabolites and cytokines such as IL-1β (16). The activation of local and recruited macrophages while important in initiating the clearance of *P. aeruginosa* also generates an environment with large amounts of the metabolic byproducts ROS and succinate.

Although the activation of the inflammasome and IL-1β release are major components of the host reaction to *P. aeruginosa* infection, the bacteria appear to thrive despite this response. Mice deficient for IL-1β receptor (IL-1R) or lacking the caspases that produce mature IL-1β exhibit decreased airway bacterial burden (17, 18). These findings suggest that these organisms have mechanisms to adapt to ROS and/or they can metabolize the succinate released from the macrophages, supporting ongoing proliferation despite host efforts of clearance through phagocytosis. Among a large number of carbon sources, succinate is a preferred substrate for *P. aeruginosa* metabolism (19–22).

### Macrophage Release of Succinate Promotes Inflammation

Succinate in the airway promotes inflammation through several mechanisms. The release of succinate from LPS-activated macrophages not only contributes to HIF-1α and IL-1β production, it also generates ROS from its own oxidation by succinate dehydrogenase (SDH) (8). ROS can damage DNA in both the host and pathogen, increasing mutation rates and the selection of variants.

Mitochondrial and cytoplasmic succinate are released into the extracellular space by cell death or by active membrane transporters of the SCL13 family (23, 24). Once extracellular, succinate signals back through its SUNCR1 receptor (also known as GPR91), which boosts IL-1β production by activated phagocytes (25). How specifically the IL-1β-IL-1R signaling favors *P. aeruginosa* pulmonary infection is under active investigation. Substantial IL-1β release and induction of phagocyte pyroptosis may cause diminished bacterial engulfment and killing, facilitating extracellular bacterial proliferation. Constant IL-1β production in the site of infection is associated with more recruitment of pro-oxidant neutrophils, which can cause local tissue damage and cell dysfunction. IL-1β signaling, tissue fibrosis and activation of chronic mechanisms of cell repair (26, 27) are pathways associated with *P. aeruginosa* infection, as seen in CF and COPD. *In vitro* macrophages studies with the intracellular pathogen *Mycobacterium tuberculosis* have shown that IL-1β-IL-1R signaling restricts intracellular bacterial proliferation by inducing glycolysis (28). *P. aeruginosa* seems to evade this pathway, as it escapes from intracellular killing by inducing IL-1β-mediated autophagy (29), changes associated with reduced glycolysis (30). These studies suggest that pulmonary Gram-negative pathogens have evolved different intracellular survival mechanisms to exploit IL-1β signaling to cause disease. Thus, a major component of the innate immune response to *P. aeruginosa*, namely the release of succinate and IL-1β, may actually have beneficial consequences for these bacteria by forcing them to adapt and survive. Accumulating data suggest that host immunometabolites have a central role in the adaptation of virulent *P. aeruginosa* to enable chronic airway infection, as commonly seen in COPD and CF.

### *P. aeruginosa* Type Three Toxins Target Host Defenses

In addition to LPS, there are many other *P. aeruginosa* gene products that activate innate immune signaling. The T3SS toxins are expressed by organisms initiating infection and have been specifically associated with fulminant pneumonia (2). The T3SS has been closely linked to the pathogenesis of acute pneumonia and the secreted virulence proteins termed effectors have multiple mechanisms that interrupt normal host metabolism, barrier and immune functions. Following pilin-mediated attachment, T3SS toxins such as exoenzyme S (ExoS) function to alter the polarization of the epithelial cell, subverting the actin cytoskeleton and perturbing epithelial tight junctions to enable bacterial invasion (31). ExoS, along with ExoT are synthetized as a bacterial response to exogenous oxidative stress and suppress neutrophil function by in turn inhibiting anti-bacterial ROS production. Both toxins affect Ras and PI3K, two key cell regulators involved in glycolysis and the generation of energy (32, 33) and cytoskeleton dynamics (34). ExoS ADP-ribosylates the signaling protein Ras, impairing its interaction with PI3K and the complex required to generate ROS by phagocytic NADPH-oxidase. ExoT also reduces PI3K function by ADP-ribosylation contributing to the deactivation of neutrophils. Thus, the activities of these toxins reflect a general theme that *P. aeruginosa* itself alters host metabolic activity which, in turn, enhances their ability to persist in the lung.

The T3SS toxins also inhibit epithelial repair, a process that diverts host metabolic functions. ExoT deregulates cell cytokinesis (35), a key process to repair damaged tissue that entails cytoskeleton remodeling and requires a substantial ATP expenditure derived from glycolysis (36). In addition to harboring an ADP-ribosyltransferase function in its C-terminus, ExoT is bifunctional and contains a GTPase-activating domain in its N-terminal portion. ExoT targets RhoA using its GTPase domain and syntaxin-2 using its ADP-ribosyltransferase domain in epithelial cells, cytoskeleton-coupled factors that direct early and late cytokinesis, respectively. Negative effects of ExoS and ExoT on pulmonary integrity not only affect epithelial cells, but also neutrophils, which undergo apoptosis following ExoT intoxication. The toxic effects of both factors are related to mitochondrial disruption as well-given that epithelial cells treated with ExoT and ExoS-producing *P. aeruginosa* strains display enrichment of Bax and Bim proteins in the mitochondrial outer-membrane, which triggers membrane instability, cytochrome C release into the cytosol and activation of pro-apoptotic caspases 9 and 3 (37).

ExoU, a patatin-like phosphatase, and ExoY also belong to the family of T3SS effectors secreted by *P. aeruginosa* during initial infection and their expression has been linked to mortality in ICU infections (2). When translocated into its target cell, ExoU displays a strong phospholipase A2-like activity, process that interferes with immune surveillance. When injected into neutrophils, ExoU induces cell death, suppressing local activity of host defenses (38). ExoU-mediated neutrophil eradication favors extracellular bacterial replication, making ExoU a key determinant for initial infection (39). ExoU is highly immunostimulatory, triggering epithelial cell proinflammatory signaling which contributes to ongoing lung damage by inducing more neutrophil recruitment, cell death and debris (40–42). ExoY, predicted to display adenylate cyclase activity within host cells, interacts with F-actin filaments to synthesize various cyclic nucleotide monophosphates (43). The intricate role between adenylate cyclase and cytoskeleton dynamics are well-described in tissue repair (44), suggesting that ExoY might also disrupt airway parenchymal barrier function by targeting cell division and viability.

Active production of ExoS, ExoT, ExoU, and ExoY is regulated in part by ArtR, a novel and unique ABC ATPase recently discovered in *P. aeruginosa* (45). ArtR represses T3SS activities, as seen by overexpression of ExoS, ExoT, and ExoY in *artR* knockout strains. ArtR also downregulates the expression of ExsA, a member of the AraC-type DNA binding protein and transcriptional inducer of type III effectors. The relationship between type III secretion and a soluble ATPase regulator indicates a direct connection between bacterial bioenergetics and the production of virulence determinants that promote inflammation, cell death and immune deregulation.

In clinical studies analyzing the expression of T3SS by *P. aeruginosa*, between 75 to 90% of strains associated with acute pneumonia and fatalities displayed functional type III secretion systems. Of these, approximately two-thirds secreted ExoS, one-third secreted ExoU, and nearly all secrete ExoT (46, 47). In contrast, a study of 56 CF patients chronically infected with *P. aeruginosa* only 12% had isolates that secreted ExoS, ExoT but not ExoU (48). When compared with environmental, urine, endotracheal, blood and wound specimens, only CF sputum isolates of *P. aeruginosa* downregulated the expression of specific T3SS products (49). These studies clearly indicate that specific families of *P. aeruginosa* gene products, such as the effectors of the T3SS, are expressed at different times during the course of infection. Of note, downregulation of the T3SS in *P. aeruginosa* has been associated with upregulation of a different secretion machinery called the type six secretion system (T6SS) which promotes adaptation to the airway environment and chronicity (50). Strategies to block the T3SS toxins while likely effective for acute pneumonia, are unlikely to disrupt established infection as occurs in CF.

### *P. aeruginosa* Adaptation to Immunometabolites—Formation of Biofilms in Response to Oxidant Stress

In contrast to the potent immunostimulatory capabilities seen in the clinical isolates of *P. aeruginosa* isolated from acute infections, typical of environmental and laboratory strains of *P. aeruginosa*, chronic isolates have acquired a very different immunogenic profile (Figure 1B). These clinical isolates have substantial decreases in the expression of components that activate the inflammasome, such as flagella, T3SS, and LPS, while up-regulating the expression of the extracellular polysaccharides that function as oxidant traps (51, 52). Similar findings have been found with other patient cohorts, where isolates from chronically infected patients with CF fail to induce inflammasome activation or IL-1β secretion due to LPS mutations or changes in T3SS toxin expression (53). The ability to follow longitudinal isolates of *P. aeruginosa* from CF patients provides insights into the process of bacterial adaptation to the inflamed airway. *P. aeruginosa*, either isolated from CF patients or simply grown in high succinate *in vitro*, have a conserved pattern of metabolic adaptation that helps them deal with the oxidant stress generated by obligate succinate metabolism.

Succinate metabolism causes sufficient oxidant stimulus to drive *P. aeruginosa* metabolic adaptation and biofilm production (54). By inducing *crc*-directed catabolite repression on glucose metabolism, succinate promotes glucose diversion into extracellular polysaccharides production (Figure 1C). ROS itself, as well as surface contact and its detection by WspR, are potent stimuli for the production of the master biofilm regulator cyclic-di-GMP (c-di-GMP), which drives synthesis of extracellular and anti-oxidant polysaccharides (55–57). ROS production contributes to bacterial stress, generating mutants better able to produce these biofilms (58). The diversion of a substantial amount of energy consumption to the production of extracellular polysaccharides imposes a metabolic cost upon the organism. Numerous features of biofilms enhance the persistence of bacteria that generate extracellular polysaccharides. The bacteria fall into a low replicative rate, a metabolic state that protects them against antibiotics that target bacterial division as mechanism of action. Adjacent bacteria form glycan-glycan interactions, developing a community of antibiotic resistant organisms able to evade phagocytosis. This biofilm metabolic program is much more restricted than the one used by planktonic bacteria, shutting-off ATP-dependent flagellar motility until becoming sessile (59, 60). Non-motile *P. aeruginosa* interact and form molecular bridges with nearby surfaces and other bacilli. Available succinate is preferentially consumed as regulated by the catabolite repressor locus *crc*, which obligates *P. aeruginosa* to consume succinate before any other carbon source present in the environment (21). While restricting flagellar swimming, *crc*-directed succinate assimilation augments twitching motility by *P. aeruginosa*, changes that favor bacteria-surface interactions and biofilm development (19, 20). The repression *crc* exerts on glucose catabolism leads *P. aeruginosa* to produce energy mainly from succinate oxidation, dedicating glucose monomers to the copious synthesis of extracellular polysaccharides (Figure 1C).

*P. aeruginosa* can form several distinct biopolymers that form biofilm. Alginate, a complex of O-acetylated D-mannuronate and its C5′ epimer L-guluronic acid, is responsible for the mucoid phenotype of *P. aeruginosa* typically isolated from patients with CF. Psl and Pel are associated with biofilm formation in many clinical settings, including pneumonias and CF. Psl is made of D-mannose, L-rhamnose, and D-glucose residues (51). Although Pel structures have been less studied, preliminary studies indicate that Pel is formed by acetylated galactosamine and glucosamine sugars (52). These polysaccharides act as traps for oxidant molecules, protecting *P. aeruginosa* from membrane and protein damage induced by activated phagocytes.

High levels of succinate are sufficient to stimulate the upregulation of *P. aeruginosa algD*, the gene that synthetizes the alginate precursor GDP-mannuronate (61, 62). Mucoid and small colony variants (SCV) of *P. aeruginosa* recovered from the CF airway display increased levels of *algD* that is induced by succinate (6, 54, 63–66). The biosynthesis of the precursors of the exopolysaccharides are supported through the glyoxylate shunt, an alternative to the TCA cycle that generates less oxidant stress and is upregulated in succinate-exposed *P. aeruginosa*. In *P. aeruginosa*, the glyoxylate shunt is controlled by *aceA* and *glcB*, two genes that produce glyoxylate first and then malate and succinate, respectively. The glyoxylate shunt saves two carbon atoms that are normally lost as CO_2_ during the TCA cycle, which are put back into circulation as malate and succinate. Malate is a gluconeogenic precursor that replenishes the bacterial cytoplasm with glucose, a precursor for the extracellular polysaccharides (Figure 1C). Succinate, is an additional glyoxylate shunt byproduct, that sustains oxidative metabolism, causing a feedback response to increase the generation of pro-biofilm mutants by upregulating the same glyoxylate shunt and by generating mutations in the DNA. The oxidative metabolism of succinate accumulated in the airway both fuels *P. aeruginosa* proliferation and drives adaptive changes to mitigate the accompanying generation of oxidants.

### Succinate Is Increased in Inflammation and in CF

The release of succinate by activated macrophages is a central component of the innate immune response to LPS in general and to *P. aeruginosa* specifically. Although succinate release is a consequence of excessive inflammation and a likely factor in many airway infections, it is a favored substrate for *P. aeruginosa* metabolism, preferentially utilized before other substrates (19, 21, 22, 67). The overwhelming predilection of *P. aeruginosa* for succinate utilization helps to explain the increased susceptibility of the CF airway for this specific pathogen, and not other opportunists that are not similarly hard wired to consume succinate before all other substrates (22). Associated with the diminished abundance of CFTR in epithelial and immune cell membranes, there is also decreased PTEN, the phosphatase and tensin homologue deleted on chromosome 10 (68). For normal PTEN function it must be tethered to CFTR at the cell membrane. PTEN regulates class I PI3Kα/β activity. As PTEN counterbalances PI3K activity, it is a central regulator of cellular metabolism. Impaired PTEN function, as occurs when there is insufficient binding to CFTR, results in the accumulation of succinate (54, 69, 70) (Figure 1D). Thus, infection in the CF airway generates even greater amounts of succinate than in normal hosts.

Not only does PTEN regulate cellular proliferation, it also induces the anti-inflammatory function of Akt and the PI3Kγ/δ subunits in phagocytes (68, 71, 72). These are proteins located in the inner leaflet of the plasma membrane during LPS-TLR4 interaction. In CF cells, the lack of the CFTR-PTEN interaction unleashes the inflammatory response, increasing NF-kB and NLRP3 activation and resulting in increased production of IL-1β and succinate (54, 68–70) (Figure 1A). PTEN deficiency in mitochondrial membranes further impairs *P. aeruginosa* killing and ultimately its clearance from the murine airway (68). The milieu of the CF airway, with decreased CFTR-PTEN interaction, increased succinate and oxidant generation provide an environment that favors *P. aeruginosa* proliferation and generates selective pressure for the organisms best suited for this environment, those protected from oxidants by their formation of biofilm (Figure 1D).

### LPS Changes in Host-Adapted *P. aeruginosa*

Analysis of the genotypic and phenotypic changes found in *P. aeruginosa* strains isolated from chronic pulmonary infections provides important insights into the tremendous versatility of these strains. Host adapted bacteria alter the expression of major structural components. LPS is consistently downregulated or altered in *P. aeruginosa* from CF patients (5). Strains from chronically infected CF patients have shorter O-antigen branches compared with control laboratory strains (73), changes predicted to have an impact on the host inflammatory response (74, 75). These isolates display reduced lateral LPS ramifications, and accumulate mutations in genes that produce these changes. In *P. aeruginosa*, LPS is synthetized by several operons involved in assembly, trafficking, and anchoring of LPS on the outer-membrane (76, 77).

LPS on the cell surface provides membrane stability (78). The process of LPS exposure is well-regulated and mediated by a set of periplasmic and outer-membrane transporters within the Lpt family of proteins. LptD, the last transporter that exposes mature LPS on the cell surface (77), has been shown to be reduced and mutated in CF *P. aeruginosa* isolates (54). This has repercussions in terms of immune-activation, as LPS-deficient strains fail to induce IL-1β production and succinate release from phagocytes. As the organisms are already in a succinate-replete environment in the inflamed CF airway, positive selection for strains that do not further add to the abundance of succinate and the oxidants associated with its metabolism may help to explain the predominance of the LPS mutant strains. Similarly, selection for mutants or variants lacking immunogenic pathogen-associated molecular patterns (PAMPs), the T3SS effectors, and flagella would also substantially decrease inflammasome activation, IL-1β and further succinate release.

### *P. aeruginosa* Modulate Host Immune Signaling

The analysis of gene expression changes in *P. aeruginosa* over the course of infection in CF, as compared with acute pneumonias has provided numerous insights into the pathogen-host dynamics. The milieu of the CF airway, with high succinate and abundant carbon sources for bacterial growth provides ample opportunity for *P. aeruginosa* colonization and adaptation, despite the presence of an immune system that functions normally in other sites. As the recruitment of phagocytes, their activation by bacterial LPS and other PAMPs induces the release of immunometabolites, such host responses drive the selection of highly adapted opportunists from the airway microbiome. These host-adapted strains suppress the expression of genes that further fuel the release of succinate, and increase the expression of extracellular polysaccharides (biofilm) to dissipate oxidant stress. In a hospital setting, with antimicrobial pressure and impaired mucociliary clearance in many hospitalized patients, these factors create conditions that favor the selection of *P. aeruginosa* and other opportunists with the metabolic flexibility to proliferate, despite the presence of an apparently robust immune response.

## *Staphylococcus aureus* Cause Acute and Chronic Infection

In contrast to *P. aeruginosa, S. aureus* are both a ubiquitous component of the commensal flora and a major cause of acute pneumonia and chronic airway infection. *S. aureus* colonize the nose in 30–40% of individuals but can cause serious infection by using numerous virulence factors and mechanisms to subvert or evade the immune system. Similar to *P. aeruginosa, S. aureus*, particularly methicillin-resistant strains including USA300 LAC that were especially prevalent a decade ago, are an important cause of severe acute pneumonia (79). The toxins produced by *S. aureus*, such as α-hemolysin (Hla) and PVL (Panton Valentine Leukocidin) contribute to the necrotizing pneumonias. Although substantial efforts in infection prevention and control have helped to decrease the incidence of those severe pneumonias, *S. aureus* strains, both sensitive and resistant to methicillin (MSSA and MRSA), are still frequently associated with airway infection, most often with a less fulminant course. *S. aureus* remain a major pathogen in COPD and especially in CF, where they are associated with chronic airway infection and are at least as common as *P. aeruginosa* (80, 81).

## Selection of SCVs in Clinical Settings

**Figure 2:**
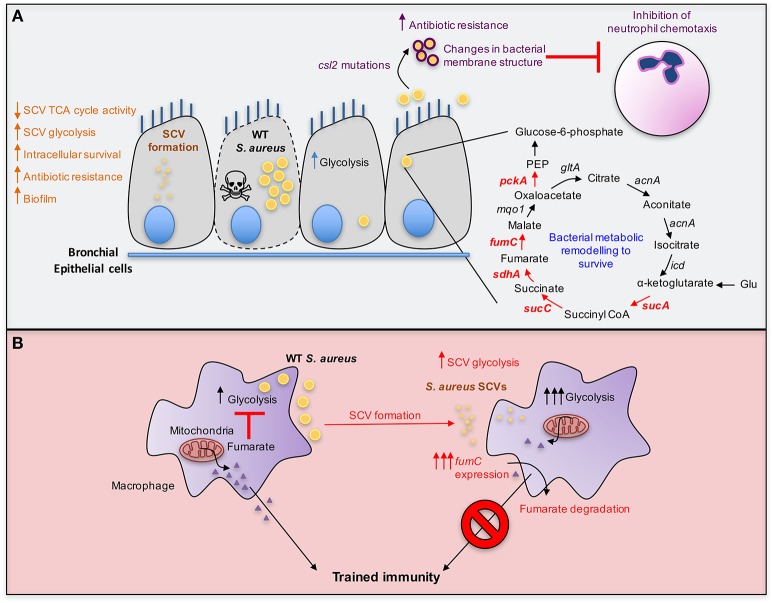
Metabolic adaptation by *S. aureus*. **(A)** *S. aureus* acquire mutations in genes associated with terminal electron transport chain components resulting in the formation of small colony variants (SCVs) that display reduced metabolism. The decrease in TCA cycle activity from SCVs results in lower electrochemical gradient which is required for the uptake of aminoglycosides, thus rendering SCVs antibiotic resistant. In addition to their metabolic adaptation, SCVs evade the host immune system by intracellular survival and have increased biofilm forming ability that protects from oxidative stress. *S. aureus* also undergo bacterial metabolic remodeling resulting in population heterogeneity following internalization by bronchial epithelial cells. One bacterial subset replicates and induces host cell death and the other slows down its growth rate, relying on the catabolism of amino acids such as glutamate to supply oxaloacetate by fueling the TCA cycle and gluconeogenesis (red arrows). *S. aureus* also acquire mutations in the gene *csl2* that results in enhanced cardiolipin synthesis and reduced phosphatidyl glycerol, thereby altering bacterial membrane composition and impairing antibiotic penetration. The reduction in phosphatidylglycerol also leads to inhibition of neutrophil chemotaxis. **(B)** *S. aureus* SCVs, such as those auxotrophic for heme, adapt to local fumarate accumulation by overexpressing *fumC* to degrade it. This helps sustain glycolysis given the role of fumarate as a glycolytic inhibitor. Fumarate degradation is also detrimental to the host and abrogates trained immunity, promoting recurrent infections.

A clinically important adaptive change to the lung by *S. aureus* is the selection of small colony variants (SCVs) from chronically infected tissues (82). SCVs were first characterized by Proctor et al. (83) as phenotypically distinct from other *S. aureus* colonies, often with pinpoint colony size that is overlooked in clinical microbiology laboratories. Although suggested to have diminished expression of toxin genes, this may not necessarily be the case *in vivo* whereby the SCVs can revert to the WT phenotype due to availability of components they are auxotrophic for (84). SCVs have a variety of mutations typically in genes associated with electron transport chain components such as menadione, and heme (82). As a result, SCVs often have altered metabolism from normal colony variants, with decreased tricarboxylic acid (TCA cycle) activity and oxidative phosphorylation (OXPHOS) and increased glycolysis to meet their energy requirements (85). SCVs are often found within host cells or in biofilms, where they are protected from antibiotics, antibodies, complement activation, and phagocytes (Figure 2A). Clinical studies in CF patients indicate that their recovery correlates with poor outcomes (86), likely influenced by the antimicrobial resistance of these variants and their ability to evade immune clearance mechanisms.

## *S. aureus* Evade Inflammasome—Mediated Clearance and Induce Necroptosis

*S. aureus* are typically considered an extracellular Gram-positive pathogen. However, these pathogens can also persist within host cells, either epithelial, endothelial or immune cells. The intracellular niche provides protection for staphylococci from some antibiotics, antibody and complement, inducing the host to utilize alternative clearance mechanisms. The induction of host cell death is often beneficial as it eliminates the intracellular niche of the pathogen and triggers an innate immune response. Bacterial pathogens can either evade host cell death or benefit from it. Like *P. aeruginosa, S. aureus* activate the NLRP3 inflammasome using several virulence factors including pore-forming toxins and phenol-soluble modulins (PSMs) (87). This normally results in pyroptotic host cell death and the release of IL-1β and bacterial clearance in the skin (88). However, NLRP3 inflammasome signaling does not clear staphylococci from the lungs of infected mice given that WT and *Nlrp3*^−/−^ mice had similar bacterial burdens following *S. aureus* intratracheal infection (89). *S. aureus*-induced NLRP3 inflammasome activation via Hla toxin prevents staphylococcal clearance by recruiting mitochondria away from phagosomes containing internalized *S. aureus* (90). In the absence of the close association of mitochondria from internalized staphylococci, macrophages were unable to effectively kill *S. aureus*. Staphyloccocal killing by co-localization with mitochondria was mediated by altered electron transport chain activity leading to ROS production, endosomal acidification and local caspase-1 activation (90).

*S. aureus*, like *P. aeruginosa*, undergo adaptive changes *in vivo*, such as the selection of SCVs. While suspected of deficient toxin production, the SCVs nonetheless induce robust immune signaling (84). *S. aureus* and even the *S. aureus* SCVs induce necroptosis, a caspase-independent mechanism of programmed cell death. Necroptosis is induced by the phosphorylation of the mixed lineage kinase domain-like (MLKL) protein by receptor-interacting protein kinase 3 (RIPK3) resulting in MLKL oligomerization at the plasma membrane and pore formation. Necroptosis does not efficiently clear the staphylococcal infection caused by either WT or SCV strains. A similar intracellular bacterial burden was recovered following infection of bone marrow-derived macrophages (BMDMs) from WT mice and *Mlkl*^−/−^ mice that are unable to initiate necroptosis (84, 91). Furthermore, necroptosis contributes to the persistence of SCVs in a mouse model of infection; SCVs failed to establish infection in *Mlkl*^−/−^ mice compared to WT mice, suggesting that necroptosis actually facilitates ongoing infection by SCVs at the expense of the integrity of the host (84). Whether this is also the case in a mouse model of pulmonary infection remains to be determined. However, *S. aureus* SCV induction of host cell death through necroptosis clearly enables the microorganisms to persist, at the expense of the integrity of the host.

## *S. aureus* Biofilm Formation Functions in the Evasion of Innate Clearance Mechanisms

Wild type strains of *S. aureus* have multiple adaptive strategies to enhance persistence in the respiratory tract. *S. aureus* readily form bacterial communities encased in biofilms, a form of protection from antibiotics, antibodies, complement activation and phagocytic clearance as well as from oxidative stress. While staphylococcal biofilms are most commonly associated with infection of indwelling intravascular devices, these Gram-positive bacteria form biofilms within the airways. From the initial stages of deposition in the airway spaces, the organisms clump together forming tight bacterial clusters that are not readily dispersed (92). *S. aureus* biofilm formation can be induced via distinct pathways including via extracellular polysaccharides or the accumulation of extracellular DNA (eDNA) and proteins (93). *S. aureus* contain two gene clusters encoding enzymes essential for two distinct polysaccharides including the polysaccharide intercellular adhesin (PIA) encoded by the *icaADBC* operon (94, 95) and the capsular polysaccharide (CP) encoded by the *cap* operon (96). *S. aureus* from CF patients have been shown to be primarily positive for PIA and negative for CP (97). A link between *S. aureus* metabolic activity and subsequent biofilm formation was suggested by the observation that the mitochondrial metabolite fumarate promotes biofilm formation in clinical isolates from CF patients *in vitro* (98). However, the mechanism of action for this metabolite-driven bacterial adaptation has not yet been determined.

Biofilm formation provides substantial protection against phagocytic clearance. Neutrophils are essential in clearing *S. aureus* via the oxidative burst and through formation of antimicrobial neutrophil extracellular traps (NETs). Unlike planktonic *S. aureus*, sessile staphylococci in biofilms remain viable even after exposure to neutrophils. Of the many mechanisms to evade bacterial killing conferred by biofilm, is the elimination of neutrophils via the release of the toxins PVL and HlgAB that induce formation of NETs (99). Whilst NETs are effective at clearing planktonic bacteria, the high concentrations of NETs produced during biofilm formation instead cause neutrophil necrosis, preventing bacterial clearance (99). Macrophages, particularly the classically activated M1 macrophages, also help clear staphylococcal infections via the production of nitric oxide and ROS. Staphylococcal biofilms skew macrophage responses towards an anti-inflammatory state M2 to evade macrophage-induced killing (100). Thus, it seems likely that staphylococcal biofilm formation impacts host metabolism and the efficiency of immune clearance mechanisms that are operative in the airways.

## Genomic Changes in Clinical Isolates of *S. aureus* From Persistent Infection

Molecular epidemiology studies have been tremendously important in tracking *S. aureus* epidemiology, and especially the global outbreaks of MRSA infection (101, 102). Less well-studied are the changes that occur in clones that have adapted to specific sites of infection, as these can occur at the genomic, proteomic, or metabolic levels. Several studies of clinical *S. aureus* strains isolated from pneumonia, in normal hosts as well as in CF patients, indicate mutations in the *agr* locus (103). The *agr* locus divergently regulates expression of *S. aureus* toxins, including Hla and several others (104) and surface proteins, the microbial surface components recognizing adhesive matrix molecules (MSCRAMMS), including protein A (105). Hla is critical in the pathogenesis of acute pneumonia, targeting A Disintegrin and Metalloprotease 10 (ADAM-10) and initiating invasive infection (106). Hla is one of several staphylococcal pore-forming toxins, capable of inducing K^+^ efflux and activating the NLRP3 inflammasome (89) to stimulate caspase-1 activation and IL-1β release. As *S. aureus* accumulate, *agr* functions in quorum sensing through the activity of small RNAs and increases Hla expression while decreasing the production of surface proteins that interact with host immune cells, such as protein A which binds both the Fc component of IgGs and the TNF receptor (107). In clinical isolates, *agr* dysfunction is associated with decreased expression of toxins, immunosuppression and staphylococcal adaptive changes to the lung.

## *S. aureus* Activate and Respond to Host Metabolism

Genomic studies indicate that the same colonizing bacteria can cause invasive infection, indicating that host-associated factors trigger the expression of genes that promote active infection, as opposed to passive colonization. The bacteria recovered from invasive infections, such as those isolated from blood or lower airways, can also regulate their gene expression to cause chronic instead of fulminant pneumonia. While the staphylococcal genes associated with the regulation of the numerous gene products that activate host immune responses, such as *agr, sar*, and many others are well-characterized, much less well-understood are the host signals that trigger global changes in gene expression that promote chronic colonization vs. invasive infection. The natural history of *S. aureus* infection in CF provides an opportunity to understand how *S. aureus* changes its transcriptional program to cause chronic infection. Analysis of gene expression in longitudinal isolates of *S. aureus* from CF patients illustrates patterns that, like *P. aeruginosa*, indicate a process of adaptation to host immunometabolites and ROS (98). Just as immune cells alter their metabolism upon microbial stimulation, *S. aureus* alters its metabolic activity, often benefiting from the metabolic reprogramming of the immune cells.

## Metabolic Adaptation of *S. aureus* to the Lung

One of the strategies employed by *S. aureus* to adapt within the human host is bacterial metabolic remodeling which is driven by the host response to infection. Following invasion of human bronchial epithelial cells, *S. aureus* adapt to the intracellular environment. Internalized bacteria display population heterogeneity with one bacterial subset replicating and eventually causing host cell death whilst another subset persists by reducing growth rates (Figure 2A) (108). In order to survive and persist, the latter subset increases fermentation and amino acid catabolism, upregulating enzymes including 2-oxoglutarate dehydrogenase (SucA), succinyl coenzyme-A synthetase (SucC), succinate dehydrogenase (SdhA), and fumarase/fumarate hydratase (FumC), which are involved in the TCA cycle activity (Figure 2A, red arrows). Other changes include increased levels of phosphoenolpyruvate (PEP) carboxykinase (PckA) involved in gluconeogenesis (108). Of note, the upregulation of these enzymes was previously shown to be important particularly in glutamate catabolism to supply intermediates such as oxaloacetate, α-ketoglutarate and succinyl-coenzyme-A and subsequently gluconeogenesis via PckA when glucose is depleted (Figure 2A) (109). This is consistent with the repression of amino acid catabolism by glucose and the inability of *S. aureus* to utilize short chain fatty acids as carbon sources, owing to the lack of β-oxidation and glyoxylate shunt pathways (109). Thus, it is important to recognize that patterns of *S. aureus* metabolism established under controlled laboratory conditions, are unlikely to accurately simulate the abundance of multiple carbon sources within the human airway. Attempts to mimic the complex milieu of the infected CF airway with “artificial sputum” provide mucins and DNA to a complex mix of amino acids and other carbon sources (110). However, even this media lacks the contribution of the immune cell metabolites and ROS that are likely to influence *S. aureus* metabolism.

## Metabolic Competition Between Host and *S. aureus*

While *S. aureus* infecting the airway are adapting to the local environment, a similar process of metabolic adaptation occurs in host cells. In response to internalized staphylococci, human bronchial epithelial cells increase glucose uptake and catabolism, as well as amino acid utilization (108). These metabolic changes observed in bronchial epithelial cells upon *S. aureus* infection are reminiscent of other studies using HeLa cells and more physiologically relevant cell types, such as bone marrow-derived macrophages (BMDMs) (111) and human primary keratinocytes (112). During staphylococcal infection, HeLa cells and murine BMDMs upregulate glycolysis and glutaminolysis (111). This creates a starvation response that induces autophagy in the host cell (111). However, autophagy provides both host and pathogen with nutrients that fuel bacterial proliferation.

*S. aureus* infection imposes a metabolic stress on host epithelial cells by competing for glucose and O_2_, which results in the activation of HIF-1α and glycolysis, as well as the many genes that are activated by this transcription factor including IL-1β (112). The *S. aureus* SCVs are especially dependent upon glycolysis, as they often lack terminal components of the electron transport chain. These SCVs associated with chronic infection substantially upregulate glycolysis in human keratinocytes and THP-1 macrophage-like immune cells (84), indicating that a hallmark of *S. aureus* infection is the induction of glucose metabolism in epithelial and immune cells. Several studies indicate that metabolically active *S. aureus* strains compete with the host for glucose to induce signaling, as opposed to non-viable organisms (112, 113), which unveils a metabolic mechanism of immune deregulation. These studies illustrate the importance of using viable bacteria for studies of host-pathogen interactions, as the metabolic signaling by both host and pathogen greatly affects immune responses.

## SCV-Induced Fumarate Degradation Inhibits Trained Immunity

An example of the importance of bacterial as well as host metabolic activity in pathogenesis is the observation that *S. aureus* from clinical settings, including the SCVs, have significantly increased *fumC* activity, up to 10,000-fold as compared to a USA300 MRSA control strain (84). The ability to degrade fumarate has several benefits for the bacteria. In addition to limiting the accumulation of fumarate, which itself inhibits glycolysis (114), fumarate induces epigenetic changes in macrophages that are associated with trained immunity (115). Trained immunity refers to the increased non-specific protection against a secondary infection at the site of the primary challenge. Fumarate inhibits KDM5 histone demethylases, promoting histone modifications such as H3K4me3, at promoters of proinflammatory cytokines, serving to enhance transcription upon secondary insult (115). The importance of *fumC* expression in the persistence of SCV infection has been illustrated using a prototypic SCV, Δ*hemB* mutant that is auxotrophic for hemin (84), as well as by using *S. aureus* clinical isolates which also showed 1,000–100,000-fold *fumC* upregulation (84, 116). Fumarate catabolism correlated with the inhibition of protection from a secondary staphylococcal challenge in a mouse model of skin infection (116) and increased *fumC* activity by SCVs resulted in lower levels of fumarate during infection of human macrophage-like cells (THP-1 cells) and peripheral blood mononuclear cells (PBMCs) (84). MRSA isolated from chronically infected CF patients also exhibit significantly elevated *fumC* activity, consistent with a shared response to the host (98). The observation that *S. aureus* SCVs blunt immune memory development by depleting fumarate from the environment (Figure 2B) supports the idea of dynamic metabolic competition between host and pathogen.

## Metabolic Changes Associated With *S. aureus* Respiratory Infection

In addition to data accrued from laboratory constructed *S. aureus* mutants and murine models of infection, numerous adaptive changes in *S. aureus* have been identified in clinical isolates. The longitudinal sampling of *S. aureus* strains from a CF patient provides the opportunity to follow how these bacteria change in response to the conditions in the host airway. A study of CF patients with MRSA infection demonstrated that these strains undergo substantial metabolic adaptive changes, without changes in immunogenicity (98). These host-adapted strains were as immunostimulatory when incubated with human airway epithelial cells or macrophage cell lines *in vitro*, as were wild type virulent USA300 MRSA strains (98). Although genomic analyses revealed several polymorphisms in expected genes as compared with control USA300 LAC, upregulation of specific metabolic pathways such as the increased *fumC* expression was observed (98). The key role of fumarate assimilation was further confirmed, as *fumC* deletion from *S. aureus* resulted in reduced colonization from murine lungs, indicating the importance of fumarate catabolism in pulmonary colonization (98). These high *fumC*-expressing host-adapted strains also displayed abundant biofilm formation, suggesting that by consuming fumarate they not only limited the development of immune memory, as described above, but also improved community lifestyle. In addition to increasing *fumC*, other metabolic changes seen in *S. aureus* isolates from a completely different study stemmed from several non-synonymous mutations in other metabolic genes, particularly loci involved in amino acid and carbohydrate metabolism (117). This host-adapted strain from long-standing infection in CF also exhibited increased biofilm formation, changes that correlated with acquisition of stop codon mutations in the master regulator *agr* (117). The progressive acquisition of mutations in metabolic genes and upregulation of *fumC* observed in *S. aureus* isolates suggest a continual adaptation to the metabolites produced by immune and epithelial cells, changes that also favor biofilm development.

## *S. aureus* Metabolic Adaptation Promotes Antibiotic Resistance

*S. aureus* adaptation to the host also includes the selection of antibiotic resistant strains in response to ongoing therapy. The acquisition of antibiotic resistance genes may or may not include a metabolic cost in fitness for the organisms. Isolation of *S. aureus* strains resistant to antibiotics, such as the cyclic lipopeptide and bacterial cell membrane disruptor daptomycin, are a major health concern. Recently, it has been shown that daptomycin-resistance is produced by single point mutations in the gene *csl2*, which codes for a cardiolipin synthase resulting in enhanced cardiolipin synthesis and reduced phosphatidylglycerol (118). This metabolic alteration resulted in changes in the bacterial membrane structure impairing daptomycin penetration and membrane disruption. Associated with these changes was the induction of a thwarted immune response, as strains with reduced phosphatidylglycerol promoted less neutrophil chemotaxis (Figure 2A). Antibiotic resistance, especially to aminoglycosides, is also exemplified by SCVs. While aminoglycosides are not first line agents for staphylococcal infection, they are frequently used in combination with cell wall active agents, and as preventative/prophylactic therapy in CF. Patients with long-term exposure to aminoglycosides have been shown to develop SCVs. The reduced susceptibility of SCVs to antibiotics is multifactorial: their intracellular location may protect them from the inhibitory concentrations, and their slow growth rate could reduce their susceptibility by non-specific mechanisms including reduced expression of pharmacological targets. Anaerobic metabolism prevents the uptake of aminoglycosides and along with reduced growth rates, OXPHOS activity and electrochemical gradients, SCVs in particular are protected from the bactericidal effects of these antibiotics (119, 120).

## Conclusions

*P. aeruginosa* and *S. aureus* are highly successful pulmonary pathogens that are able to undergo adaptive changes to first colonize mucosal surfaces, elude mucociliary clearance to cause acute infection, and then adapt to the metabolic demands of the airway to establish chronic infection. These organisms have substantial metabolic flexibility to adapt to the environment within the host often by acquiring mutations or by changing their transcriptional profiles. While the phenotypic changes associated with adaptation to the lung, such as the expression of extracellular polysaccharides, loss of motility, or selection of small colony variants are readily apparent, most of the other adaptive changes are not. Vaccination or even therapeutic strategies to target these organisms must first consider if the intent is the prevention of colonization or suppression of organisms that have already undergone substantial adaptation to persist in the airways.

Whereas the downstream effect of these adaptive changes to establish chronic infections has been thoroughly studied, what drives these changes is less well-understood. Indeed, recent studies are focusing on the metabolic cross-talk between the host cell and the pathogen as well as the response of the pathogen to a milieu that is often filled with immunosignaling metabolites. Despite the abundance of immunometabolites, further studies are required to better understand their role in bacterial pathogenicity in physiologically relevant settings.

## Author Contributions

SR, TW, and AP wrote the entire manuscript and produced figures.

### Conflict of Interest

The authors declare that the research was conducted in the absence of any commercial or financial relationships that could be construed as a potential conflict of interest.
